# Reinstating the Novelty P3

**DOI:** 10.1038/srep31200

**Published:** 2016-08-11

**Authors:** Robert J. Barry, Genevieve Z. Steiner, Frances M. De Blasio

**Affiliations:** 1 Centre for Psychophysics, Psychophysiology, and Psychopharmacology, Brain & Behaviour Research Institute, and School of Psychology, University of Wollongong, Wollongong NSW 2522, Australia; 2 The National Institute of Complementary Medicine, School of Science and Health, Western Sydney University, Penrith NSW 2751, Australia

## Abstract

P300 (or P3) is a major positive complex in the human event-related potential, occurring some 300 ms after stimulus onset, and long thought to be the cortical correlate of the Orienting Reflex, our automatic attention-grabbing response to a novel stimulus. The Novelty P3 was the third P3 subcomponent discovered (after P3a and P3b) and appeared promising in its sensitivity to stimulus novelty, the defining characteristic of the Orienting Reflex. But some 15 years later it was claimed to be indistinguishable from the previously-discovered P3a. This led to a decline in interest in the field and confused nomenclature, with some studies using “P3a” and “Novelty P3” interchangeably. However, recent similar studies have again reported three subcomponents of the P3. Further, using single-stimulus habituation paradigms, in addition to P3a and P3b, a later decrementing P3 subcomponent has been reported, and recently labelled “HabP3” to avoid contention. We report three studies to resolve this chaotic situation, arguing for identification of the late subcomponent following the P3a and P3b as the Novelty P3. Reinstatement of the Novelty P3 as the central index of the Orienting Reflex will have widespread impact in a range of theoretical, practical, and clinical areas involving novelty processing and attention.

While the autonomic Orienting Reflex (OR) has provided a wealth of information in regard to our understanding of basic learning mechanisms, and fostered theory development, the search for a cortical correlate of this reflex has been long and difficult. It was proposed more than a quarter of a century ago that the P300 (or P3) event-related potential (ERP) component held the most promise as a cortical index of the OR^1^. The P3 is a large centro-parietal deflection, peaking approximately 300 ms after stimulus onset, which increases as the degree of certainty concerning stimulus probability decreases^2^. Initial reports emphasised the need for attention as a necessary condition for elicitation^2^,^3^,^4^,^5^. However, evidence of a late positive wave to unpredictable but irrelevant stimuli^4^,^6^, differing in both latency and topographic distribution from that originally described^2^, suggested that the P3 represented a complex that differed with paradigm rather than being a single entity, leading to its label as the “late positive complex” (LPC)^7^.

An early (220–280 ms) fronto-central P3a elicited by both attended and non-attended deviant stimuli in an oddball task (with high probability standard stimuli and low probability “oddball” deviants) was then distinguished from a later (310–380 ms) parietal P3b that was enhanced for attended deviants^8^. Squires and colleagues also reported a broadly-distributed late Slow Wave (SW) contemporaneous with the P3a and P3b peaks^8^.

A third P3 subcomponent, a *later* (360–450 ms) frontal Novelty P3 elicited by rare nontargets interspersed randomly in an attended oddball series (the “Novelty Oddball” paradigm) was also reported in the mid seventies^9^. From our perspective, the Novelty P3 is of particular interest – it is the most likely cortical correlate of the OR, being uniquely associated with stimulus novelty, the OR’s defining parameter.

P3a, P3b, Novelty P3, and SW overlap and sum to form the LPC^10^, and a major problem is how to disentangle these. In recent years there has been an increase in the use of principal components analysis (PCA) for this purpose^10^,^11^,^12^,^13^,^14^,^15^. This is a variety of factor analysis, and groups ERP activity into factors that correspond to ERP components, each reflecting topographically-similar time-locked activity.

In an influential study, Simons *et al*.^13^ replicated Squires *et al*.’s^8^ paradigm and an auditory Novelty Oddball from Courchesne *et al*.^16^. ERP averages for the 220–420 ms epoch from each of the two data sets (*N* = 9 each) were combined in a PCA using the covariance matrix; further details were not provided. Four factors were extracted from each set. Exploration of the two factor structures showed that the distributions of what were identified as the P3a factor scores and the Novelty P3 factor scores were statistically indistinguishable, leading the authors to assert that “the arguments originally marshalled in support of the distinction between the Novelty P3 and the P3a have not been empirically supported”^13^. This has become the dominant perspective in the literature^17^,^18^.

However, the issue does not appear to be totally settled. For example, some researchers state the equivalence between Novelty P3 and P3a, but continue to use “Novelty P3”^19^,^20^. In this context it is particularly interesting to note that some recent papers have reported two components in the Novelty P3/P3a^19^,^21^. These studies largely use peak measures rather than PCA, and have reported two Novelty P3s in a passive auditory novelty oddball task: a central distribution in the 185–285 ms latency range, and a diffuse later (285–385 ms) subcomponent^19^,^20^. A recent study^21^ pursued this separation in an attended novelty oddball and a three-tone oddball (but calling the peaks “P3a” rather than “Novelty P3”), and used temporal PCA followed by spatial PCA to separate what they termed the early and late P3a subcomponents. Using poststimulus epochs from 200 to 475 ms, they reported major temporal factors at ~250 and 350 ms. The first of these was identified as a new “early P3a”, essentially replicating the results of a passive paradigm^19^,^20^ in active tasks. The second factor was separated by spatial PCA into a frontal P3a and a parietal P3b.

Our interest in this issue arose with our first PCA study of the LPC in a long-ISI habituation paradigm^10^. We presented 15 stimulus trains, each containing 5 standards, a deviant tone, and a re-presentation of the standard. ERPs were averaged across trains for each position in the train. A temporal PCA found four LPC components, identified as P3a, P3b, Novelty P3 and SW. The Novelty P3 for the standards decremented with repetition, recovered at the change trial, and dishabituated at the re-presentation of the standard – the classic markers of habituation^22^. Our subsequent studies at long ISIs have also reported both P3a and the Novelty P3^23^,^24^,^25^,^26^,^27^. In an effort to avoid contention, we recently labelled our Novelty P3 as “HabP3”^25^,^26^. In all our studies^10^,^23^,^24^,^25^,^26^,^27^, the four components have appeared in the same temporal order – P3a, P3b, HabP3/Novelty P3, and SW – suggesting a stable subcomponent structure within the LPC in habituation paradigms.

The inconsistencies evident in this brief review of the LPC literature highlight the need for, and the significance of, the current paper. We report three studies aiming to clarify confusion regarding the early/late P3a/Novelty P3, particularly in relation to our findings of a third P3 component (HabP3/Novelty P3) and the stable sequencing of the three LPC subcomponents in long-ISI habituation paradigms. Our first study is a replication of a recent study by McDonald *et al*.^21^ to establish a data set containing evidence for the reported early/late P3a/Novelty P3. Our second study explores a wider range of components in this data set, allowing more components than has been typical in prior investigations^13^,^21^. Our final study examines the impact on the components found in Study 2 when including participant data from an additional task, a long-ISI Habituation paradigm, in the PCA. We expect to find persuasive evidence for the existence of three subcomponents of the LPC occurring prior to the SW: P3a, P3b and Novelty P3, in that temporal order. Our criteria for identifying the Novelty P3 are: a PCA component that is part of the LPC, but distinct from the P3a and P3b, that is sensitive to novelty in the Novelty Oddball, and reflects stimulus repetition and change in the Habituation paradigm.

## Study 1

This study was a replication of the Novelty Oddball and Three-tone Oddball dataset reported in McDonald *et al*.^21^, and followed their procedure and data analysis as closely as possible.

### Results and Discussion

In the Three-tone Oddball, across the 24 participants there were 3 omission errors (0.2%), 33 commission errors to rare nontarget distractors (2.9%), and 13 commission errors to standards (0.1%) (*M*_total errors_ = 12.0, *SD* = 1.0). In the Novelty Oddball task, there were 3 omission errors (0.2%), 21 commission errors to novels (1.8%), and 12 commission errors to standards (0.1%) (*M*_total errors_ = 16.3, *SD* = 1.2). None of these differed between the tasks: *t*_targets_(23) = 0.00, *p* = 1.000, 2-tailed; *t*_rare nontargets_(23) = 1.47, *p* = 0.156, 2-tailed; *t*_standards_(23) = 0.15, *p* = 0.880, 2-tailed. Average target response time (RT) was Three-tone Oddball: 434 ms (*SD* 55 ms) and Novelty Oddball: 425 ms (*SD* 61 ms); these did not differ, *t*(23) = −1.08, *p* = 0.292, 2-tailed.

**Figure 1:**
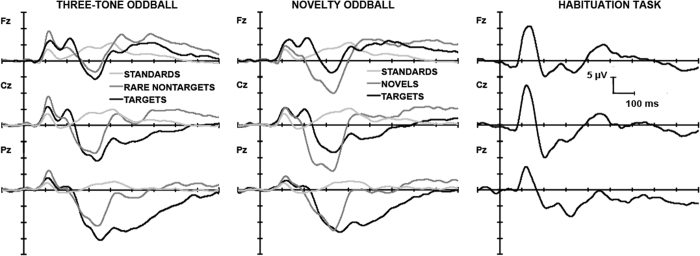
Grand mean (*N* = 24) midline ERPs for the auditory paradigms assessed. Left: Three-tone Oddball presented with 480 stimuli; 80% standards, 10% rare nontargets, and 10% targets. Middle: Novelty Oddball presented with 480 stimuli; 80% standards, 10% novel distractors, and 10% targets. Right: Habituation paradigm presented with 13–15 stimuli (10 standards, 1 deviant, 2–4 standards) and involving no task or response requirements. Only the first 11 responses were assessed here.

Figure 1 shows grand average ERPs for the stimulus types in the Three-tone Oddball (left) and the Novelty Oddball (middle). Targets in both elicited a large positive P3 with a parietal maximum. Novels in the Novelty task elicited large components of similar amplitude across the three sites. Our ERP peaks had shorter latencies (~25 ms) than those previously reported^21^, possibly reflecting subject variability and our smaller sample size, so we carried out PCA on a slightly earlier epoch of the same duration (175 to 450 ms *cf*. 200–475 ms).

**Figure 2:**
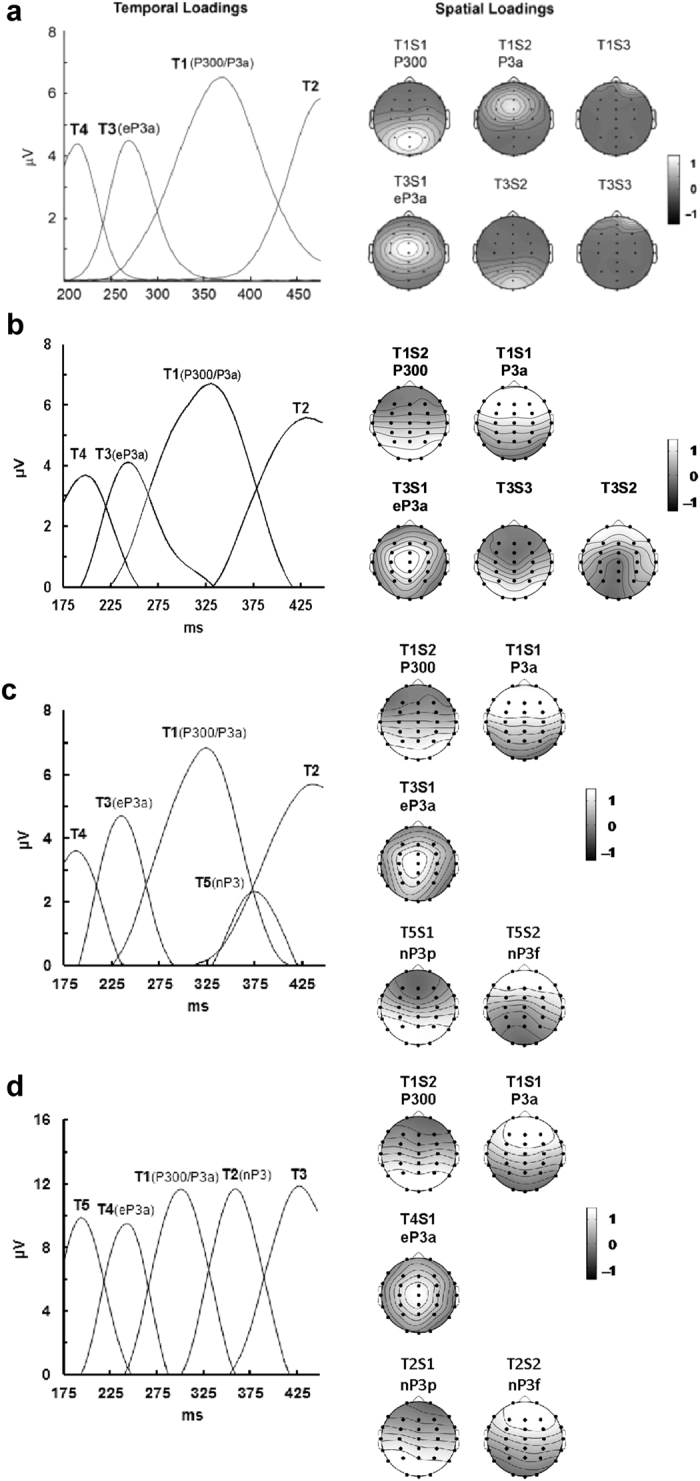
Temporal and spatial factor loadings. (**a**) Outcomes reported in McDonald *et al*.^21^, reprinted with permission. Corresponding outcomes are illustrated for the current sample (*N* = 24) derived from: (**b**) Study 1 assessing the Novelty and Three-tone Oddball datasets following McDonald *et al*.‘s^21^ protocol with a 4-factor PCA solution; (**c**) Study 2 assessing the Novelty and Three-tone Oddball datasets with a 6-factor PCA solution; and (**d**) Study 3 assessing the Novelty Oddball, Three-tone Oddball, and Habituation datasets with a 5-factor PCA solution.

Figure 2(a) shows the results of the original McDonald *et al*.^21^ study and, using the same labels for corresponding components, Fig. 2(b) shows the results of Study 1. The scaled component loadings from the temporal PCAs are presented on the left, and close similarity is evident. In our 4-factor temporal solution, T1 is the dominant component with a peak latency of 335 ms corresponding to the major target response (labelled P300/P3a), and T3 is the earlier temporal component with a peak latency of 247 ms identified as the early P3a (hereafter eP3a). These peaked some 35 and 24 ms earlier than the corresponding McDonald *et al*.^21^ components. Moreover, they carried 49% and 13% of the variance, respectively, almost identical to the corresponding values reported by McDonald *et al*.^21^ (49 and 14%).

A 2-factor spatial PCA of T1 resulted in factors consistent with the original findings^21^ – a parietal P300 (T1S2) and a fronto-central P3a (T1S1), but with their order reversed; compare spatial loadings, right Fig. 2(a,b). Furthermore, our first of three spatial factors derived from T3 had a central topography comparable to McDonald *et al*.‘s^21^ eP3a. That is, we reproduced in our current dataset the three temporo-spatial ERP components previously identified^21^.

Table 1 displays the results of our statistical analyses and their correspondence to those published in McDonald *et al*.^21^. We found that P300 (T1S2) showed a significant main effect of stimulus category and a significant task by stimulus category interaction. For the Three-tone Oddball, this factor was larger for targets when compared to rare nontargets and standards. Scores for standards and rare nontargets did not differ. In the Novelty Oddball, target and novel scores were significantly higher than those for standards, but novel and target scores did not differ significantly. Overall our P300 results were mostly consistent with those of McDonald *et al*.^21^, the only discrepancy being that there was no significant main effect of task for P300 as indicated in Table 1; this was most likely driven by our comparable factor scores for rare nontargets (Three-tone Oddball) and novels (Novelty Oddball).

For the P3a (T1S1), there was a significant effect of task, stimulus category, and task by stimulus interaction. For the Three-tone Oddball, factor scores for all stimulus categories were low and did not differ. In the Novelty Oddball, the P3a factor was significantly larger for novels than both standards and targets. Factor scores for standards and targets did not differ in the novelty task. That is, all effects for P3a were replicated.

eP3a (T3S1) showed significant effects of task and stimulus category, and a task by stimulus interaction. For the Three-tone Oddball, there were no significant effects of stimulus category. For the Novelty Oddball, factor scores for novels were significantly greater than for targets and standards, which did not differ. Table 1 shows that all effects for eP3a were replicated.

Our replication of McDonald *et al*.^21^ was carried out with the stimuli and stimulus sequences used in the original paper, and generated PCA components that essentially replicated the original results. Note that we followed as closely as we could all steps in data processing and PCA component extraction and analysis. One aspect of the PCA approach not mentioned by McDonald *et al*.^21^ is the issue of selecting the appropriate number of components to extract in the PCA; we explore this issue in Study 2.

## Study 2

In Study 1 we selected our components following the default procedure in Dien’s PCA toolbox^28^, which generated a close replication of the previous results^21^. Here we explored a wider range of components in the same data set. Our question is: what happens if we select more temporal components than previously chosen? Temporal PCA is a data-summary device that, in the ERP context, aims to summarize and group waveform data over the input time points into a smaller number of components exhibiting topography and peak latencies that match the original data. There have been many criteria proposed to establish the optimal number of factors to extract. Dien and Frishkoff^29^, the PCA source cited in McDonald *et al*.^21^, advocated a modified version of Cattell’s Scree test^30^, which looks for steps in the descending variance pattern. In this parallel test^31^, the scree slope of the data is compared with a scree slope from noise estimated by inverting alternate ERPs to cancel all but noise; components carrying more variance than noise are retained. It has been noted that this gives a lower bound to the number of factors, and that retention of more factors may improve the analysis^29^.

### Results and Discussion

With a 6-component temporal PCA, an additional component appeared in the latency window of the LPC. With further increases in the number of components, the first five remained stable. T5 from the 6-component solution was a candidate for identification as the Novelty P3. Accordingly, we submitted temporal components T1, T3, and T5 to spatial PCA with Promax rotation. Figure 2 shows our loadings and component topography from Study 1 (b) and Study 2 (c) for the components of interest. The temporal loadings in Fig. 2(c) indicate that T5, carrying 6% of the variance, emerged after the P300/P3a with a peak latency of 379 ms. Components T1 to T4 can be seen to have retained latencies similar to those in Study 1, but are slightly narrower and better defined. In this PCA, T1 carried 45%, and T3 carried 13% of the variance (*cf*. 49% and 13% in Study 1). The temporo-spatial topographies shown on the right of panel c (*cf.* panel b) indicate that the spatial components T1S2 (P300) and T1S1 (P3a) appear unchanged, as does T3S1 (eP3a); as in Study 1, these were derived from 2- and 3-factor spatial solutions, respectively. In a 3-factor spatial solution, the new T5 showed a parietal T5S1 that we term here the parietal Novelty P3 (nP3p), and a frontal T5S2 that we term the frontal Novelty P3 (nP3f). Table 2 displays the results of our statistical analyses and their correspondence to those in Study 1.

Statistical effects in the two tasks noted for the three temporo-spatial factors identified in Study 1 were essentially duplicated in this PCA decomposition, as indicated in the last column of Table 2. The single exception was in T1S2, McDonald *et al*.’s^21^ P300, where the previously significant target > rare nontarget effect in the Three-tone Oddball dropped in probability from *p* = 0.049 to *p* = 0.083.

For the nP3p (T5S1), there was no effect of task, a marginal stimulus category effect, and a significant task by stimulus interaction. For the Three-tone Oddball, the nP3p was significantly larger for targets and rare nontargets relative to standards; scores for targets and rare nontargets did not differ. In the Novelty Oddball, the nP3p factor was significantly larger for targets and novels than standards, and for targets than novels.

For the nP3f (T5S2), significant main effects of task and stimulus category were observed, with a task by stimulus category interaction. For the Three-tone Oddball, there were no significant differences between targets and rare nontargets, but responses to each were larger than those to standards. For the Novelty Oddball, factor scores for targets were greater than for standards, and those for novels were greater than both targets and standards.

Including more components in the temporal PCA prior to rotation uncovered an additional component following the dominant P300/P3a. This component remained stable if further components were extracted, and does not appear to have impacted on the previously-identified components generated in Study 1 – they retained similar latencies, topography, and dependencies on stimulus type. The additional component, identified as the Novelty P3 (nP3), was found to have two spatially-defined subcomponents, a parietal nP3p and a frontal nP3f. Overall, the nP3p was large to targets and rare nontargets in the Three-tone Oddball, and to targets and novels in the Novelty Oddball, but was not uniquely enhanced to novelty. In contrast, the nP3f spatial factor was significantly larger in the Novelty Oddball than the Three-tone Oddball, and larger to novels than targets in the Novelty Oddball. This suggests that the frontal nP3f is the major Novelty P3 spatial factor, meeting the criteria required for such a component.

These data indicate that a Novelty P3, temporally distinct from the P3a and the P300, can be found in these paradigms if an adequate PCA is used. Undue restriction of the number of components in the PCA will exclude its observation. This should not be surprising, as the problem with the optimal number of components to be extracted has long plagued this field, and indeed, the author of the PCA toolkit^28^ warned about such an outcome^29^.

## Study 3

But what of the Novelty P3 in habituation paradigms? We were interested in whether a combined PCA on data from the Novelty Oddball, Three-tone Oddball, and the Habituation paradigm would produce a set of components similar to those described in Study 2 for the two oddball paradigms, and if the components found would show correspondence to our previous^10^,^23^,^24^,^25^,^26^,^27^ temporal PCA reports of P3a, P3b, and nP3, most of which^23^,^24^,^25^,^26^,^27^ were based on single-trial ERPs from similar habituation studies.

### Results and Discussion

The mean ERPs at the major midline sites in the Habituation paradigm are shown on the right in Fig. 1. There was a large fronto-central N1 around 120 ms, and a P2 near 200 ms. These were followed by a P3 complex before a SW near 450 ms. A five-component temporal PCA of the combined data from the three paradigms yielded the loadings shown in the left of Fig. 2(d). Some temporal components can be identified from the ERPs: P2 (T5 at 199 ms), and SW (T3 at 431 ms). By analogy with Studies 1 and 2, we can label T4 (at 247 ms) as eP3a, T1 (at 307 ms) as P300/P3a, and T2 (at 363 ms) as nP3. These P3 components carried 14% (eP3a), 22% (P300/P3a), and 22% (nP3) of the variance; the increased contribution of the nP3 component (from 6% to 22% of the variance) indicates the impact of the Habituation ERPs. The spatial loadings on the right in Fig. 2(d) show a parietal T1S2 and frontal T1S1 that match those in Studies 1 (Fig. 2b) and 2 (Fig. 2c), again derived from a 2-factor solution. The T4S1 obtained here from a 4-factor solution appears very similar to the T3S1 found in our McDonald *et al*.^21^ replication (Study 1; Fig. 2b). The two spatial Novelty P3 subcomponents, a parietal nP3p (T2S1) and a frontal nP3f (T2S2), were found with a 2-factor solution. Overall, these appear comparable with the T5S1 and T5S2 (respectively) found in Study 2 (compare corresponding components in Fig. 2 panels c,d). Table 3 displays the results of our statistical analyses and their correspondence to those in Study 2.

Statistical effects obtained for the five temporo-spatial factors identified in Study 2 were generally duplicated in this PCA decomposition as indicated in the last column of Table 3, with the following exceptions. In P300 (T1S2), the task by stimulus category interaction disappeared. For P3a (T1S1) the marginal effect of task only approached significance. For the nP3p (T2S1) factor, the stimulus category effect was now significant, while the interaction between task and stimulus category was non-significant. In the Three-tone Oddball, nP3p responses were significantly larger for targets than rare nontargets, and in the Novelty Oddball, the nP3p factor was only marginally larger for targets than novels. Lastly, the nP3f (T2S2) responses in the Three-tone Oddball were significantly larger here to targets than rare nontargets.

**Figure 3:**
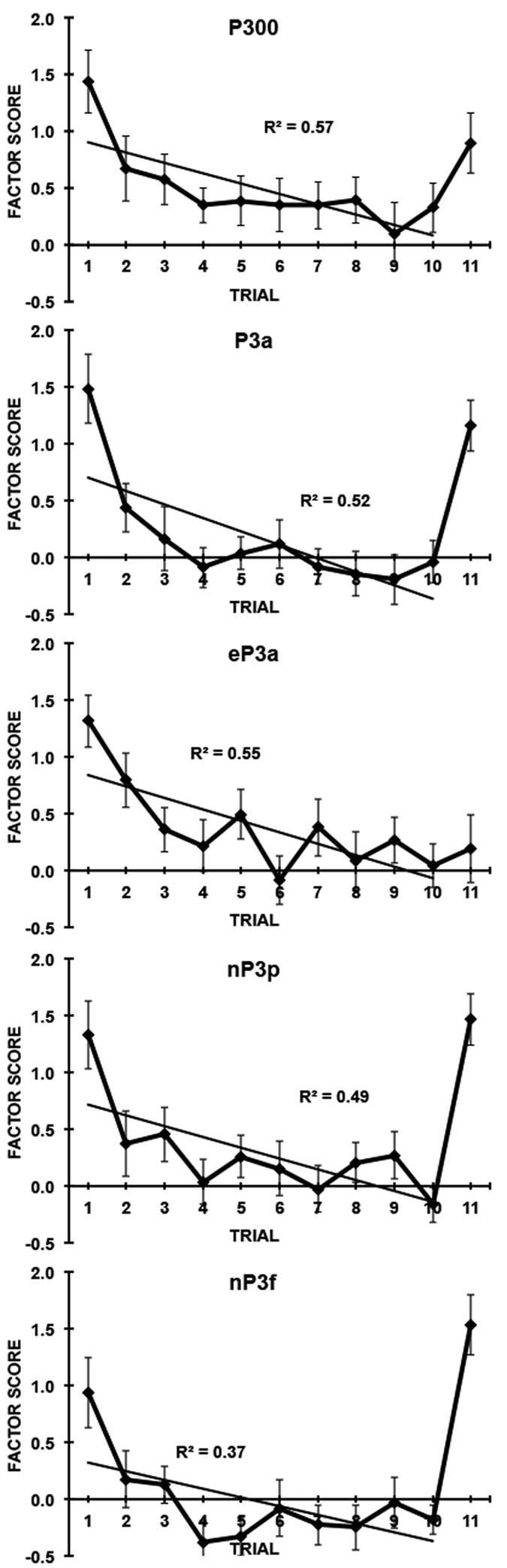
Trial effects in each of the spatial subcomponents identified in Study 3. Component factor scores for the Habituation paradigm are presented as a function of the 10 stimulus repetitions and deviant trial.

Scores across subjects for each of these subcomponents in the Habituation dataset are displayed as a function of the 10 stimulus repetitions and change trial in Fig. 3, with corresponding statistics presented in Table 4. For the parietal P300 (T1S2), decrement occurred in a linear trend over trials 1–10, but there was no significant recovery at the change. The frontal P3a (T1S1) showed linear and quadratic decrement, and a significant recovery at the change. eP3a (T4S1) also decremented in linear and quadratic trends, but failed to show recovery to the change. Lastly, both nP3p (T2S1) and nP3f (T2S2) showed decremental linear and quadratic trends over trials, and significant recovery at the change.

This long-ISI Habituation paradigm has a series of LPC components that match those found here in the Novelty Oddball and the Three-tone Oddball tasks. Using the nomenclature of McDonald *et al*.^21^, these have been labelled in temporal order as the eP3a and P300/P3a, with the addition of a subsequent nP3 component. The spatial components in Study 3 offer clear matches to those found in Study 2, and demonstrate similar patterns to stimuli in the Three-tone Oddball and Novelty Oddball paradigms. Over trials in the Habituation paradigm, decrement was shown by all 5 spatial components in linear and sometimes quadratic trends. Recovery at the change trial was found in P3a (T1S1) and the nP3 subcomponents; of these, only the nP3f (T2S2) showed a larger response to the change than the initial stimulus, fulfilling the last of our criteria, and suggesting its supremacy as the Novelty P3.

## General Discussion

Study 1 replicated the McDonald *et al*.^21^ study that first presented evidence for a new late positive ERP component in the attended Novelty Oddball task. Using exactly the same stimuli and stimulus presentation sequence, and their temporal-spatial PCA extraction procedure, we confirmed the existence of their early P3a – a separate temporal component at 247 ms – and late P3a that was a spatial subcomponent of the temporal component at 335 ms. This late component was separated by the spatial PCA into a parietal P300 and a central P3a. Our results essentially confirmed their findings.

We were concerned, however, that using the parallel test^31^ refinement of Cattell’s Scree test^30^ may still lead to the extraction of too few components, generating a poor model of the data. Hence Study 2 relaxed this criterion and ran additional temporal PCAs with more components. Each additional component extracted results in a redistribution of the variance amongst the components after rotation, but the major components reach stability quickly^32^. In our case, we found that a 6-factor temporal PCA generated an additional component in the LPC latency window, and that the first five components (including the additional LPC component) remained stable for further solutions with additional components. The new T5 component at 379 ms, later than the P300/P3a complex, reflected the properties expected of a Novelty P3. Spatial decomposition of this component evidenced a parietal nP3p and a frontal nP3f.

McDonald *et al*.^21^ failed to find a larger response to the rare nontargets than the standards in the Three-tone Oddball in any of their spatial subcomponents. In Studies 1 and 2 we confirmed the absence of this effect in the three corresponding components: P300, P3a, and eP3a. However, in Studies 2, our additional nP3p and nP3f components were both larger to rare nontargets than standards. In the Novelty Oddball, the response to novels was previously reported as greater than that to targets for P3a and eP3a, but not P300^21^; again our results replicated this in both Studies 1 and 2. Additionally, this effect was found in our nP3f component in Study 2. Together these results support the new nP3 (particularly the nP3f) uncovered in Study 2 as a good candidate for identification as the Novelty P3 in these paradigms.

We note that a similar restriction in the number of temporal components extracted probably occurred in at least one other influential study^13^. That study found similar components in data from replications of the P3a study of Squires *et al*.^8^ and the Novelty P3 study of Courchesne *et al*.^9^ – essentially a P3a and P300 in each. We contend that extraction and rotation of further components would have uncovered an additional Novelty P3 component in the Courchesne data set.

As noted earlier, the P3a peak of Squires *et al*.^8^ had a latency of about 240 ms, in sharp contrast to the P3b peak at about 350 ms. The first report of two P3a peaks, obtained from novel-minus-standard difference waves at Fz, noted an early P3a at 230 ms and a late P3a at 315 ms^33^. Although that paper did not mention the Novelty P3, it did relate the late P3a to the orienting response. Another study in relation to the early/late P3a from the same group examined a similar novel-minus-standard difference wave^34^; in that paper the early/late P3a was referred to as early/late Novelty P3. None of these robust components required spatial PCA for their separation, being apparent in the raw ERPs or difference waves. In McDonald *et al*.^21^ and our Studies 1 and 2, the major element of eP3a appears as the temporal PCA component T3. It therefore appears that a better label for the *early P3a/nP3* is simply “P3a”, reflecting the component discovered in 1975^8^. As is apparent in Fig. 2, these components appear to be framed by the P2 near 200 ms (T4 in McDonald *et al*.^21^ and Studies 1 and 2; T5 in Study 3) and the Slow Wave near 425 ms (T2 in McDonald *et al*.^21^ and Studies 1 and 2; T3 in Study 3).

Furthermore, we suggest that the components previously identified in the literature as the *late P3a*^33^ and *late nP3*^34^, and our temporal components T5 of Study 2 and T2 of Study 3, should be identified as the Novelty P3. We do not consider that the *late P3a* identified as a spatial subcomponent of the temporal P300 by McDonald *et al*.^21^, and our three Studies here, relates to the *late P3a*^33^ or the *late nP3*^34^ reported in earlier studies, or represents the Novelty P3. Rather, we consider it is simply a reproducible frontal component of the P300/P3b. This is supported by our temporal separation of nP3 from the late P3a in both Study 2 and Study 3. For ease of comparison, our proposed clarification of this nomenclature is presented in Table 5, including the suggested correspondence of components identified in prior investigations.

Study 3 examined the LPC from a long-ISI Habituation paradigm in the same subjects, adding these data into the PCA alongside data from the Oddball paradigms, and obtained the same sequence of 5 temporal components. The three P3 components were identified here as P3a (T3 in Studies 1 and 2, T4 in Study 3), P300/P3b (T1 in Studies 1–3), and nP3 (T5 in Study 2, T2 in Study 3), with a substantial increase in the temporal loading of the nP3 (see Fig. 2c,d for comparison) reflecting the increased influence of novelty in this paradigm. Decrement over 10 trials was observed with the spatial subcomponents of each of these, but recovery to a change stimulus was found in only three: the frontal subcomponent of P300/P3b (T1S1) and the frontal and parietal subcomponents of nP3. Of the latter subcomponents, only the frontal nP3 demonstrated a greater response to the novel change than to the initial stimulus, indicating that this is the optimal marker of novelty across the three paradigms tested.

We have found similar results for this third temporal P3 component in several previous habituation studies^23^,^24^,^25^,^26^,^27^, in all of which we identified the components in the latency order: P3a, P3b, and Novelty/Hab P3. This stability (confirmed here) should be sufficient to firmly establish these components in this order. For all of these^23^,^24^,^25^,^26^,^27^, subdivision into spatial subcomponents has not been found necessary for their adequate measurement. Rather, the frontocentral P3a, parietal P3b, and frontal nP3 appear robust in temporal PCAs, as well as being the dominant subcomponents identified in the temporo-spatial PCAs. That is, these three P3 components appear to be reliable in terms of their different latencies and topographies, and can be identified by simple temporal PCA of the LPC. The PCA subdivision of these components into their spatial subcomponents, as reported here and in previous studies, therefore appears somewhat unnecessary. Moreover, this spatial subdivision produces subcomponents that carry little variance, and have not been linked reliably to unique stimulus or situational parameters; thus they require extensive further investigation to establish their importance.

Our evidence appears sufficient to reinstate the Novelty P3 as a major entity in the P3 “family”. As seen in Table 5, we urge the renaming of “late P3a” as the *Novelty P3*, and the “early P3a/early Novelty P3” as just the *P3a*. Also, in the habituation literature, the label “HabP3” should be dropped in favour of *Novelty P3*. Although it is unlikely that our extensive study here will ever be replicated, we would welcome and support such an effort. Apart from this, confirmatory evidence can be sought in any of the paradigms used here, as well as in others involving novel stimuli. To this end, we also encourage care in selecting the number of components to be extracted and rotated, in order to avoid overlooking important ERP components.

We conclude that our results confirm the existence in the LPC of the traditional P3a, P3b, and Novelty P3, sequenced in that order. Our current understanding emphasises the P3a as a marker of attention to the stimulus which precedes the P3b as a reflection of decision making, particularly in relation to the stimulus or task context. Following these, the nP3 provides an index of stimulus novelty, the core feature in relation to the elicitation of the Orienting Reflex, directing attention and additional processing resources to novel stimuli. We consider that the three components potentially occur in different proportions in the ERP response to all stimuli, and that such a perspective can be helpful in understanding perceptual and cognitive processing. Most importantly, reinstatement of the Novelty P3 should encourage a new wave of research addressing the important role of the Orienting Reflex in our interactions with the environment.

## Methods

### Participants

Twenty-four undergraduate students from the University of Wollongong (mean age = 23.6, *SD* = 6.5 years, 16 females, all right handed) completed this study as one means of satisfying a research participation course requirement. None reported neurological or psychiatric illnesses, and none were taking psychotropic medication. All reportedly refrained from psychoactive substances for at least 12 hours, and from caffeine, alcohol, and tobacco for at least 2 hours. All participants had normal or corrected-to-normal vision and self-reported normal hearing.

### Ethics Statement

All experimental procedures were carried out in accordance with a protocol approved by the joint South Eastern Sydney/Illawarra Area Health Service and University of Wollongong Health and Medical Human Research Ethics Committee. Participants were informed about the experiments and told that they were free to withdraw at any time without penalty; all provided written informed consent.

### Electrophysiological Recording

Continuous EEG data were recorded DC–70 Hz with a Neuroscan Synamps 2 digital signal-processing system and Neuroscan 4.3.1 Acquire software. Data were acquired from 30 scalp sites (Fp1, Fp2, F7, F3, Fz, F4, F8, FT7, FC3, FCz, FC4, FT8, T7, C3, Cz, C4, T8, TP7, CP3, CPz, CP4, TP8, P7, P3, Pz, P4, P8, O1, Oz, O2) and A2, using an electrode cap with tin electrodes. A1 was used as a reference and the cap was grounded by an electrode located midway between Fp1, Fp2 and Fz. Stimulus presentation was controlled by a linked stimulus computer using Presentation (Neurobehavioral Systems Inc.). The electrooculogram (EOG) was recorded using tin cup electrodes placed on the outer canthus of each eye for horizontal movements, and 2 cm above and below the left eye for vertical movements. Impedance was less than 5 kΩ for all electrodes. Scalp and EOG potentials were amplified with a gain of 500 and digitally sampled at 1000 Hz.

### Procedure and Tasks

Participants completed a demographic and screening questionnaire, and were then fitted with EEG recording apparatus. They were seated in an air-conditioned room 600–800 mm in front of a 48.3 cm (19″) Dell LCD monitor, and instructed to fixate on a 10 × 10 mm grey cross centred on a black background. Acoustic stimuli were delivered binaurally through Sony MDR V700 circumaural stereo headphones.

Initially, participants completed an EOG/EEG calibration task^35^. They then completed three experimental tasks: a Novelty Oddball task, a Three-tone Oddball task, and a Habituation paradigm. The first two of these were counterbalanced between subjects, and either preceded or followed the Habituation paradigm. The Novelty Oddball presented a single block of 480 stimuli (48 novels: *p* = 0.1; 48 targets: *p* = 0.1; 384 standards: *p* = 0.8) using the stimuli from McDonald *et al*.^21^, all at 80 dB. The 1000 Hz standards and 2000 Hz targets were of 336 ms duration with 10 ms rise/fall times; novels were unique environmental stimuli from the New York Psychiatric Inventory^36^ and had a mean duration of 347 ms (*SD* = 53, range = 214–399 ms). Stimuli were presented with 1 s stimulus-onset-asynchrony (SOA) in the exact sequence used by McDonald *et al*.^21^ The Three-tone Oddball used the same SOA and stimulus order, with the same standards and targets, but replaced the novels with 500 Hz nontarget distractors at 80 dB, with 336 ms duration, and 10 ms rise/fall times. Participants were instructed “Your job is to respond only to the targets (the high-pitched sounds); be careful not to respond to any other sounds”, using a Logitech precision game controller with their dominant hand.

The Habituation paradigm presented a single “indifferent” block of 13–15 stimuli (with no response required): 10 standards, 1 deviant, 2–4 standards; only the first 11 responses were analysed. Stimuli were 650 and 1500 Hz tones at 80 dB, each of 336 ms duration with 10 ms rise/fall times, and a randomly-varying SOA of 5–7 s. Standard and deviant tones were counterbalanced between subjects.

### Data Quantification

EEG data were EOG corrected using the RAAA EOG Correction Program^35^. Data were extracted offline using the Neuroscan Edit software, band pass filtered (0.1–30 Hz, zero-phase shift, 24 dB/Octave), epoched for −100 ms pre- to 900 ms post-stimulus, and single trials were baselined to the prestimulus period. Epochs were manually inspected for any additional artefact, contaminated trials were excluded from analysis, and data were down-sampled to 250 Hz.

## Study 1

Average ERPs from each subject for each condition in both the Novelty Oddball and Three-tone Oddball tasks were together subjected to temporal-spatial PCA, matching those parameters reported by McDonald *et al*.^21^, including the epoch length of 275 ms. We used Dien’s PCA toolbox^28^ (v. 2.23) in MATLAB (The Mathworks, v. 8.0.0.783, R2012b), and first carried out a temporal PCA, followed by a spatial PCA of temporal factors of interest. The parallel test^31^ was used to guide our selection of the number of temporal factors to extract. All PCAs used the covariance input, and were rotated to simple structure using Promax rotation. As per McDonald *et al*.^21^, factor scores for each identified component were subjected to repeated-measures analysis of variance (ANOVA) with variables of task (Three-tone vs. Novelty oddball) and stimulus category (targets vs. rare nontargets vs. standards); rare nontargets in the Three-tone oddball were compared with Novels in the Novelty oddball. Greenhouse-Geisser corrections of degrees of freedom were applied where necessary. Paired *t*-tests were used in follow-up analyses and Bonferroni-corrected for multiple comparisons.

## Study 2

We repeated the temporal PCA stage used in Study 1, extracting 5, 6, 7 and 8 components, and rotating these sets using Promax. After a stable solution was found, that is, when the extraction of additional factors showed no noticeable impact on the major components identified, we followed this with spatial PCA, using Promax rotation of the factors of interest for comparability with Study 1.

## Study 3

In this study we included in our PCA the data from the Habituation paradigm – ERPs at each trial (1 to 11) – in addition to our data from both the Three-tone and Novelty Oddball tasks. For comparability, we used the same temporal window as in Studies 1 and 2, and followed the exploratory approach of Study 2, starting with 4 temporal factors. Once a stable solution was identified, this was again followed by spatial PCA with Promax rotation for each factor of interest.

## Additional Information

**How to cite this article**: Barry, R. J. *et al*. Reinstating the Novelty P3. *Sci. Rep.* **6**, 31200; doi: 10.1038/srep31200 (2016).

## Figures and Tables

**Table 1:** Study 1 component factor score statistics based on the identified components derived using McDonald *et al*.‘s^21^ protocol assessing the Novelty and Three-tone Oddball data.

	*F*	*p*	*ε*	Replicated?
**P300** (T1S2)				
Task	1.2	0.292		✘
Stimulus Category	20.2	<0.001	0.72	✓
Task * Stimulus Category	66.3	<0.001	0.70	✓
Three-tone (T > RN)		0.049		✓
Three-tone (T > S)		<0.001		✓
Novelty (T > S)		<0.001		✓
Novelty (N > S)		0.001		✓
**P3a** (T1S1)				
Task	5.4	0.029		✓
Stimulus Category	73.2	<0.001	0.79	✓
Task * Stimulus Category	40.6	<0.001	0.72	✓
Novelty (N > T)		<0.001		✓
Novelty (N > S)		<0.001		✓
**eP3a** (T3S1)				
Task	6.0	0.023		✓
Stimulus Category	15.2	<0.001	0.68	✓
Task * Stimulus Category	3.3	0.044	0.75	✓
Novelty (N > T)		<0.001		✓
Novelty (N > S)		0.001		✓

The last column indicates the general correspondence between the present results and those of McDonald *et al*.^21^. T = Target; RN = Rare nontarget; S = Standard; N = Novel stimuli.

**Table 2:** Study 2 factor score statistics for the components identified in the Novelty and Three-tone Oddball 6-factor PCA solution.

	*F*	*p*	*ε*	Replicated Study 1?
**P300** (T1S2)				
Task	0.8	0.368		✓
Stimulus Category	21.3	<0.001	0.71	✓
Task * Stimulus Category	62.3	<0.001	0.71	✓
Three-tone (T > RN)		0.083		✘
Three-tone (T > S)		<0.001		✓
Novelty (T > S)		<0.001		✓
Novelty (N > S)		<0.001		✓
**P3a** (T1S1)				
Task	5.0	0.035		✓
Stimulus Category	63.5	<0.001	0.80	✓
Task * Stimulus Category	41.0	<0.001	0.75	✓
Novelty (N > T)		<0.001		✓
Novelty (N > S)		<0.001		✓
**eP3a** (T3S1)				
Task	10.0	0.004		✓
Stimulus Category	24.9	<0.001	0.72	✓
Task * Stimulus Category	7.2	0.006	0.72	✓
Novelty (N > T)		<0.001		✓
Novelty (N > S)		<0.001		✓
**nP3p** (T5S1)				
Task	2.4	0.132		
Stimulus Category	2.6	0.085	0.06	
Task * Stimulus Category	43.64	<0.001	0.87	
Three-tone (T = RN)		0.263		
Three-tone (RN > S)		<0.001		
Three-tone (T > S)		<0.001		
Novelty (T > N)		0.046		
Novelty (N > S)		<0.001		
Novelty (T > S)		<0.001		
**nP3f** (T5S2)				
Task	4.7	0.041		
Stimulus Category	37.8	<0.001	0.67	
Task * Stimulus Category	9.1	<0.001	0.89	
Three-tone (RN = T)		0.130		
Three-tone (RN > S)		<0.001		
Three-tone (T > S)		0.036		
Novelty (N > T)		<0.001		
Novelty (N > S)		<0.001		
Novelty (T > S)		0.030		

The last column indicates the general consistency of these outcomes with those of Study 1, for the corresponding components. T = Target; RN = Rare nontarget; S = Standard; N = Novel stimuli.

**Table 3:** Study 3 factor score statistics for the components identified in the combined PCA including the Novelty Oddball, Three-tone Oddball, and Habituation paradigm.

	*F*	*p*	*ε*	Replicated Study 2?
**P300** (T1S2)				
Task	0.1	0.754		✓
Stimulus Category	43.3	<0.001	0.63	✓
Task * Stimulus Category	0.6	0.543	0.01	✘
Three-tone (T = RN)		0.215		✓
Three-tone (T > S)		<0.001		✓
Novelty (T > S)		<0.001		✓
Novelty (N > S)		<0.001		✓
**P3a** (T1S1)				
Task	3.7	0.07		✘
Stimulus Category	49.9	<0.001	0.64	✓
Task * Stimulus Category	22.8	<0.001	0.47	✓
Novelty (N > T)		<0.001		✓
Novelty (N > S)		<0.001		✓
**eP3a** (T4S1)				
Task	5.5	0.028		✓
Stimulus Category	18.9	<0.001	0.42	✓
Task * Stimulus Category	13.4	<0.001	0.34	✓
Novelty (N > T)		<0.001		✓
Novelty (N > S)		<0.001		✓
**nP3p** (T2S1)				
Task	1.3	0.259		✓
Stimulus Category	68.5	<0.001	0.73	✘
Task * Stimulus Category	1.1	0.329	0.00	✘
Three-tone (T > RN)		0.004		✘
Three-tone (RN > S)		<0.001		✓
Three-tone (T > S)		<0.001		✓
Novelty (T > N)		0.068		✘
Novelty (N > S)		<0.001		✓
Novelty (T > S)		<0.001		✓
**nP3f** (T2S2)				
Task	7.7	0.011		✓
Stimulus Category	45.2	<0.001	0.64	✓
Task * Stimulus Category	23.4	<0.001	0.48	✓
Three-tone (T > RN)		0.039		✘
Three-tone (RN > S)		<0.001		✓
Three-tone (T > S)		<0.001		✓
Novelty (N > T)		0.001		✓
Novelty (N > S)		<0.001		✓
Novelty (T > S)		<0.001		✓

General comparability with the outcomes of Study 2 are indicated in the last column. T = Target; RN = Rare nontarget; S = Standard; N = Novel stimuli.

**Table 4:** Study 3 statistics assessing trial effects in the Habituation paradigm subcomponent factor scores across the 10 stimulus repetitions and change trial.

	*F*	*p*	*ŋ*^*2*^_*p*_
**P300** (T1S2)			
Trial 1–10 (Linear)	21.9	<0.001	0.49
Trial 1–10 (Quadratic)	3.7	0.065	0.14
Trial 10 < 11	2.3	0.142	0.09
Trial 11 vs. 1	2.4	0.128	0.10
**P3a** (T1S1)			
Trial 1–10 (Linear)	17.4	<0.001	0.43
Trial 1–10 (Quadratic)	15.3	0.001	0.40
Trial 10 < 11	14.9	0.001	0.39
Trial 11 vs. 1	1.1	0.298	0.05
**eP3a** (T4S1)			
Trial 1–10 (Linear)	18.1	<0.001	0.44
Trial 1–10 (Quadratic)	12.9	0.002	0.36
Trial 10 < 11	0.2	0.698	0.01
Trial 11 < 1	11.6	0.002	0.33
**nP3p** (T2S1)			
Trial 1–10 (Linear)	12.7	0.002	0.35
Trial 1–10 (Quadratic)	5.4	0.030	0.19
Trial 10 < 11	36.5	<0.001	0.61
Trial 11 vs. 1	0.17	0.682	0.01
**nP3f** (T2S2)			
Trial 1–10 (Linear)	9.4	0.005	0.29
Trial 1–10 (Quadratic)	14.5	0.001	0.39
Trial 10 < 11	41.9	<0.001	0.65
Trial 11 > 1	5.8	0.025	0.20

Trial 1–10 = response decrement; Trial 10 < 11 = response recovery; Trial 11 vs. 1 = response recovery vs. initial response.

**Table 5:** Our proposed nomenclature for the three P3 components (P3a, P3b, and Novelty P3) and the corresponding labels used by other work cited throughout this paper.

Proposedomenclature	P3a		P3b		Novelty P3 (nP3)	
*Prior*	*Label*	*Peak (ms)*	*Label*	*Peak (ms)*	*Label*	*Peak (ms)*
Squires *et al*.^8^	P3a	220–280	P3b	310–380	—	—
Courchesne *et al*.^9^	—	—	—	—	novels P3	360–450
Simons *et al*.^13^	P3a/Novelty P3	~260–280	P3b/P300	~330–360	—	—
McDonald *et al*.^21^	Early P3a (eP3a)	271	P300	370	—	—
Barry *et al*.^23^	P3a	229	P3b	315	Novelty P3 (nP3)	397
MacDonald *et al*.^25^	P3a	264	P3b	309	HabP3/nP3	371
Escera *et al*.^33^	Early P3a	230	—	—	Late P3a	315
Yago *et al*.^34^	Early Novelty P3 (Early nP3)	235	—	—	Late Novelty P3 (Late nP3)	335
Current Paper (Study 2)	P3a	239	P3b	327	Novelty P3 (nP3)	379
Current Paper (Study 3)	P3a	247	P3b	307	Novelty P3 (nP3)	363

Labels and peak latencies for the three components derived from Studies 2 and 3 in the current manuscript are detailed in the final two rows for comparison.
